# Tumor heterogeneity reshapes the tumor microenvironment to influence drug resistance

**DOI:** 10.7150/ijbs.72534

**Published:** 2022-04-24

**Authors:** Aiping Zhang, Kai Miao, Heng Sun, Chu-Xia Deng

**Affiliations:** 1 Cancer Center, Faculty of Health Sciences, University of Macau, Macau SAR, China.; 2 Centre for Precision Medicine Research and Training, Faculty of Health Sciences, University of Macau, Macau SAR, China.; 3 MOE Frontier Science Centre for Precision Oncology, University of Macau, Macau SAR, China.

**Keywords:** tumor heterogeneity, tumor microenvironment, drug resistance

## Abstract

Tumor heterogeneity is one of the hallmarks of cancer and a challenge in the field of oncology. Tumor heterogeneity is the main cause of drug resistance, leading to therapeutic failure. Mechanically, tumor heterogeneity either directly affects therapeutic targets or shapes the tumor microenvironment (TME) by defining transcriptomic and phenotypic profiles to influence drug resistance. Tumor heterogeneity evolves spatially and temporally during tumor development, leading to the constant reprogramming of the TME. Advances in molecular profiling technologies and precision oncology platforms have allowed us to uncover the impact of tumor heterogeneity on drug resistance in the context of the TME. In this review, we focus on the processes during which genomic mutations drive tumor heterogeneity and the mechanisms through which tumor heterogeneity reprograms the TME to affect drug resistance and patient prognosis.

## Introduction

Drug resistance is the leading cause of therapeutic failure in cancer patients. The biological determinants of drug resistance include tumor growth kinetics, tumor burden, tumor heterogeneity, physical barriers, immune system, tumor microenvironment (TME), undruggable genome, and therapeutic pressures. Among these determinants, tumor heterogeneity is the main issue that causes drug resistance 1-4. Drug resistance caused by tumor heterogeneity exists across all cancer types and all therapeutic modes, including chemotherapy, radiotherapy, targeted therapy, and immunotherapy. The therapeutic targets, cancer cells themselves, and TME are the three major components that determine how well a drug works.

Tumor heterogeneity describes cellular population diversity between tumors of the same type in different patients (intertumor heterogeneity) or even within a single tumor (intratumor heterogeneity) 5. Generally, tumor genetic mutations, transcriptional alterations, protein level changes, and epigenetic modifications of these cellular characteristics all manifest tumor heterogeneity 5. In addition to these intrinsic factors, some other extrinsic factors, such as pH, hypoxia, and crosstalk between tumor cells and other stromal cells within the TME also affect tumor genotypes and phenotypes, further leading to tumor heterogeneity 5. Tumor heterogeneity exists ubiquitously across all cancers. Chromosome mutational evolution, together with ecosystem pressures, leads to temporal and spatial segregation of mutation clones and subclones. In a pan-cancer study involving 2658 human cancer genomes spanning 38 cancer types, mutation patterns, such as single nucleotide variations (SNVs), indels, structural variations (SVs), copy number alterations (CNAs), subclonal drivers, subclonal selections, and mutation signatures, were extensively characterized 6. The authors observed that subclonal expansion occurred in nearly 95.1% of samples, and different cancer types showed cancer-specific genetic tumor heterogeneity patterns 6. At the single-cell level, tumor heterogeneity is also characterized by the analysis of the single-cell copy number in neuroendocrine prostate cancer 7, the analysis of gene expression in non-small-cell lung cancer (NSCLC) 8, hepatocellular carcinoma 9-11, brain cancer 12, breast cancer 13, kidney cancer 14, head and neck cancer 15, and so on.

As mentioned above, the therapeutic targets, cancer cells themselves, and the TME are a three-component system of tumor heterogeneity leading to drug resistance. The TME comprises not only cancer cells but also the complicated tumor stroma, which mainly includes structures such as the basement membrane, extracellular matrix, vasculature, and various other types of cells (immune cells, fibroblasts, endothelial cells, etc.) 16-18. During tumor progression, tumor heterogeneity continuously reshapes the TME by changing the transcriptional levels of some genes to make a more complicated TME, which causes divergence of trajectory development, immune landscape, intercellular networks 8, 19 and so on. Under different circumstances, the interactions among cells present diversity in the TME. Wu et al. 8 observed divergent and complex intercellular networks, which revealed a comprehensive view of the TME cellular network, including the following aspects: angiogenesis, immune activation, and suppression, as well as cancer-associated fibroblasts (CAFs). These interactions often determine tumor development and drug treatment efficacy, which includes drug resistance and prognosis 20. Here, we review the genomic changes that drive tumor heterogeneity formation, development, and drug resistance in the context of tumor heterogeneity-induced TME reprogramming.

## Genomic processes promote tumor evolution and tumoral heterogeneity

Tumor evolution is defined as the biological progression of tumor cells, which usually begins from a particular cell(s) in normal tissue, gradually forming a tumor ecosystem after accumulating massive mutations, where some of these mutations serve as the fuels and drivers of their host cells to undergo clonal expansion. Genetic mutation, selection, and drift are the three components of tumor evolution, where genetic mutation generates new variation, selection and drift, leading to clonal expansion and contraction 21. In principle, tumor evolution has four putative models: linear, branching, neutral, and punctuated. For each model, tumor evolution accompanies clone selection 22. Clonal selection is the most important of these three components 21. Basically, tumor-selective pressure comes from two sources. On one hand, the pressure is exerted by TME elements such as immune cell response, hypoxia, pH alteration, and limited nutrients. On the other hand, the pressure comes from external causes: tumor therapy activities, such as conventional chemotherapy, and immunotherapy leading the clone selection. Random genetic drift describes the frequency changes of a gene variant (allele) due to random death and birth in a population, which may cause alleles to increase, reduce and/or disappear 23. It is known that during the tumor evolution process, directed by diverse selection pressure, one small tumor cell population may evolve into a dominant clone or trunk driver 24. By analyzing 2500 tumors of different types, it was found that 91% of these tumors had at least one driver mutation, and on average, there were 4.6 drivers in each individual tumor, which indicates pervasive variation diversity across cancers 25. Additionally, the tumor evolution model could switch from one to another or even coexist multiple different models in a particular tumor. The relationships between two subclones can be sibling (branching) or parent-child subclones, and the percentages of sibling subclones are approximately 3 times greater than those of parent-child subclones 6.

**Figure 1:**
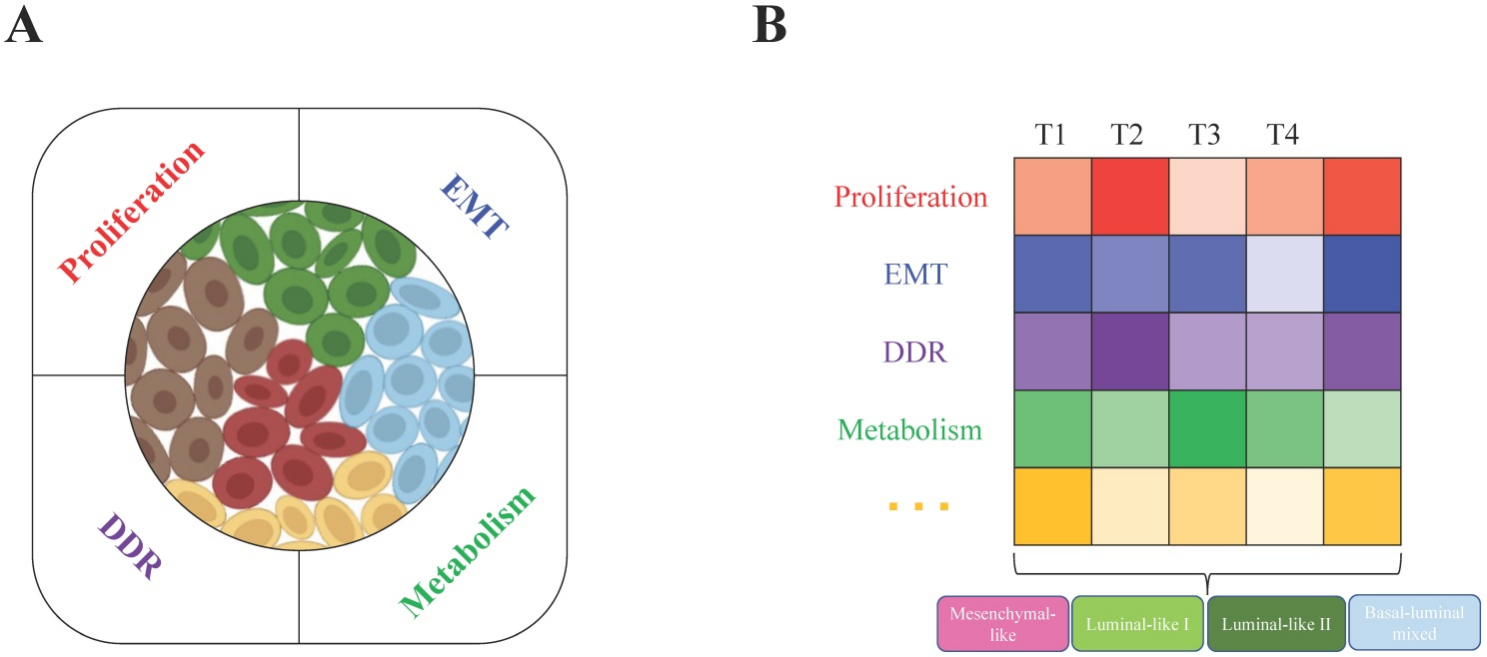
Intratumor and intertumor heterogeneity of BRCA1-deficient mammary tumors. A. Single-cell RNA sequencing revealed intratumor heterogeneity, and the main contributing factors (proliferation, EMT, metabolism and DDR) were identified by the expression patterns of feature genes in BRCA1-deficient mammary tumors. B. Based on histopathological characteristics and whole transcriptome profiles, the four subtypes mesenchymal-like, luminal-like I and II, and basal/luminal mixed tumors were classified in heterogeneous BRCA1-deficient mammary tumors. Further analysis found that the above contributing factors might be the main determinants that caused intertumor heterogeneity (T1, T2, T3, and T4 indicate different tumors).

All these biological characteristics and processes accelerate tumor evolution and increase tumor heterogeneity, which may depend on increased genome instability. For example, in breast cancer, BRCA1/2 loss of function is a primary driver. BRCA1 deficiency leads to genome instability, which results in tumor heterogeneity at the genetic level, such as DNA copy number alterations in many genes, such as Myc, Met, Pten, and Rb1 26. Not only distinct mutations are generated and accumulated in different tumor cells within a tumor (intratumor level), but the genetic alteration profiles are also very diverse among different tumors (intertumor level). With the help of single-cell RNA sequencing (scRNA-seq) technology, in one of our recent studies, we found that the heterogeneous subgroups within the BRCA1-deficient mammary tumors are classified mainly due to the different activities of cell proliferation, metabolism, DNA damage response (DDR) and epithelial-to-mesenchymal transition (EMT) (**Figure 1A**) 24. At the intertumor level, based on histopathological characteristics, basal and luminal lineage marker expression patterns and especially the whole transcriptome profiles, the BRCA1-deficient mammary tumors could be divided into four major subtypes: mesenchymal-like, luminal-like I and II, and basal/luminal mixed tumors (**Figure 1B**). Because a tumor is a mixture of heterogeneous tumor cells, we hypothesized that the constitution of different tumor cells (intratumor heterogeneity) mainly determines the subtype of the tumor (intertumor heterogeneity), while the complex microenvironmental cells and factors also significantly affect both the intra- and intertumor heterogeneity.

## Tumor heterogeneity drives the dynamics and diversity of the tumor microenvironment

Tumor heterogeneity, as a consequence of tumor evolution, has been considered an essential basis for reconstructing tumor evolution and other aspects of tumor exploration. It has been detected with different genetic mutation patterns, such as single nucleotide variations (SNVs), indels, structural variations (SVs), and copy number alterations (CNAs) 6, 27. Genetic and epigenetic changes cooperate to define tumor cell transcriptomic and phenotypic profiles, leading to the consistent reprogramming of the TME 28, 29. Wu et al. applied scRNA-seq to profile tumor heterogeneity and the TME in advanced non-small-cell lung cancer (NSCLC) 8. Lung adenocarcinoma (LUAD) and lung squamous carcinoma (LUSC) were well classified by the high expression of NAPSA and TTF-1 (NKX2-1) for LUAD and TP63 and CK5 (KRT5) for LUSC. Based on the differential expression of some canonical markers, lung epithelial cells comprise alveolar type 2 (AT1) cells, alveolar type 2 (AT2) cells, basal cells, ciliated cells, and club cells. Analysis of their developmental trajectory showed that LUAD cells transitioned from AT2 cells and club cells, while LUSC cells transitioned from basal cells, which showed different cancer developmental trajectories. In LUAD patients, the group with known oncogenic driver mutations enriched a cluster of macrophages with high CCL13 expression, while the group without known oncogenic driver mutations did not show this phenomenon. Additionally, intratumor heterogeneity was positively correlated with neutrophils and two subsets of macrophages and negatively correlated with plasma cells. Similarly, another recent study also performed scRNA-seq on early-stage LUAD samples that manifest as subsolid nodules (SSNs), together with primary LUAD, lymph node metastasis (mLUAD) and normal lung tissues (nLung), to decode tumor heterogeneity and the TME dynamic ecosystem 30. The multicellular ecosystem of SSN presents distinct features when compared to nLung and mLUAD. For example, NK cells, mast cells and T cells in the SSN are significantly enriched compared with those in the mLUAD, which indicates better immunosurveillance in the SSN. By analyzing stromal cell subtypes, the authors found that the fibroblast subcluster in the SSN was similar to that in the nLung, while the endothelial cells were more similar to those in the mLUAD. Therefore, they hypothesized that these two cell types were reprogrammed at different stages. Regarding cell-cell communications among TME cells, SSN showed more abundant interactions in lymphocyte recruitment and homing than mLUAD. Thus, the cellular components and crosstalk in the TME are dynamically changed as tumors progress, and consequently, at different tumor stages, the abundance and functional orientation of TME components are significantly varied.

**Figure 2:**
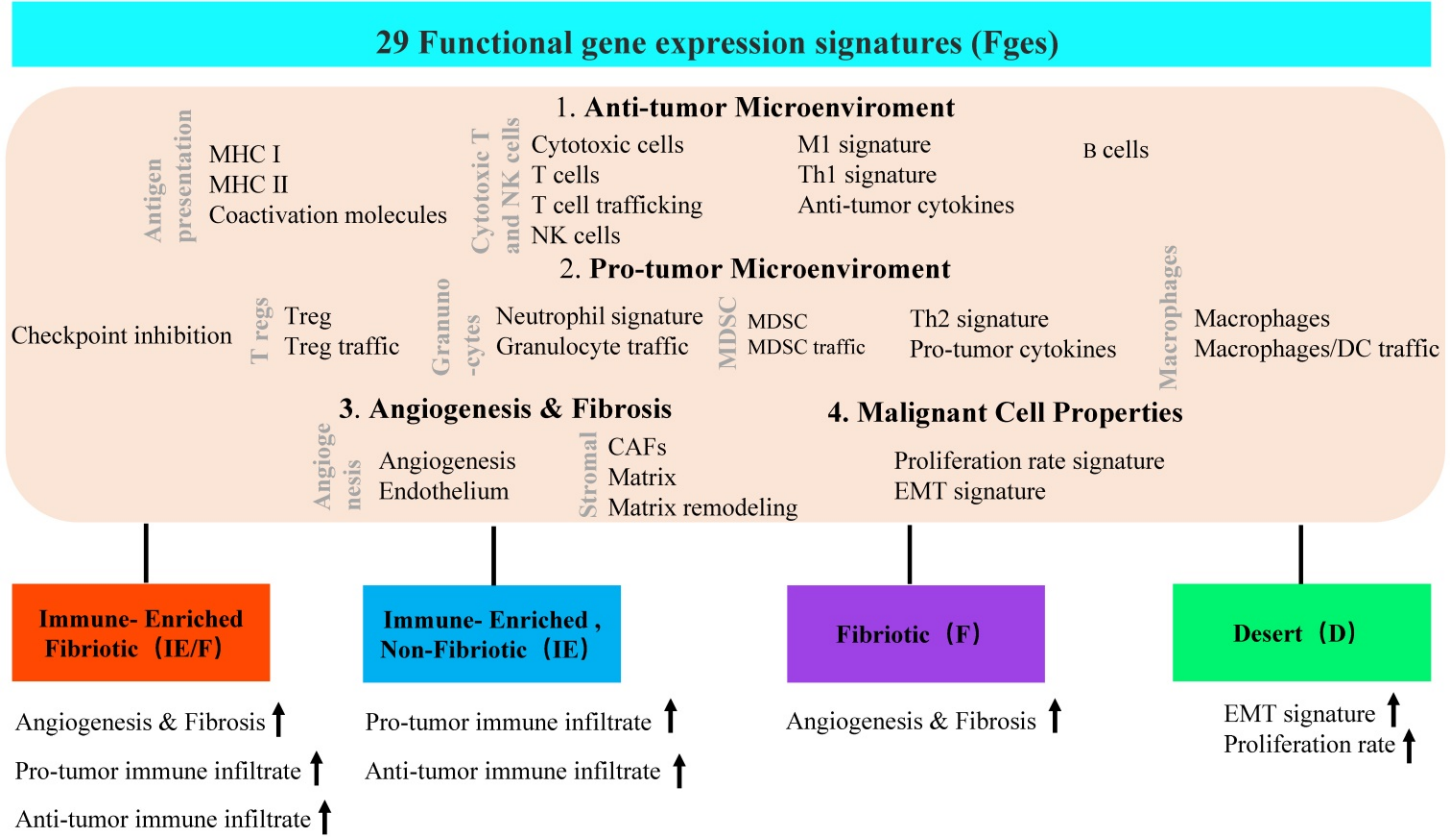
Four TME subtypes were classified by 29 functional gene expression signatures. First, 29 functional gene expression signatures (Fges) that represent major functional components and immune, stromal, and other cellular populations of the tumor were selected. Based on biological functions, these 29 Fges contained four categories: antitumor microenvironment, protumor microenvironment, angiogenesis & fibrosis, and malignant cell properties. Then, 468 TCGA cutaneous melanomas (TCGA-SKCM) were classified into four distinct TME subtypes based on unsupervised dense clustering by using those 29 Fges (the upward arrow indicates the corresponding upregulated item).

In addition to dynamic reprogramming, another property of the TME that is affected by tumor heterogeneity is TME complexity. To comprehensively characterize a tumor and its TME, researchers usually perform tumor subtype classification and try to dig out each classified subtype more deeply. A typical heterogeneous tumor type, triple-negative breast cancer (TNBC), has been well studied to dissect the complexity by classifying this tumor subtype based on different molecular levels, such as cell populations, gene expression and genetic alterations (**Table 1**). At the TME level, Bagaev et al. leveraged transcriptomic data from more than 10000 cancer patients to investigate the TME subtypes and the associations between subtypes and genomic alterations 31. Here, 29 functional gene expression signatures (Fges) defined by TME cell populations, biological processes, cancer cell properties and signaling signatures were established (**Figure 2**). By unsupervised analysis of these TME Fges, cancer patients were classified into four distinct TME subtypes conserved across 20 different cancers: 1) immune-enriched, fibrotic (IE/F); 2) immune-enriched, nonfibrotic (IE); 3) fibrotic (F); and 4) immune-depleted (D), which included significantly varied expression of 29 Fges and presented distinct features (**Figure 2**). Further analysis demonstrated that TME subtypes were associated with genomic alterations. For example, in the IE subtype, there were enriched amplifications of the immune checkpoint genes CD274 (PD-L1) and PDCD1LG2 (PD-L2); alterations in the histone modification genes CREBBP, KMT2A, and PBRM1 and regulation of the DNA mismatch repair gene MSH2; and mutations in the antigen presentation or interferon-inducible gene CASP8. However, in subtype D, amplifications were shown in the cellular proliferation genes CDK6 (8p11) and BCL2L1 and the telomere elongation regulation gene RTEL1 (20q.13). Additionally, the IE subtype has the highest number of mutations in MHC class I-related genes, while the F subtype has the lowest percentage, which results in different responses to immunotherapy.

More specifically, tumor immunity and the tumor immune microenvironment can also be researched by classifying immune subtypes. Tumor immune properties mainly consist of the subsets of infiltrating immune cells and immune-related gene expression in tumors 32. At different stages and conditions during tumor development, immune contexture significantly varies due to constant reprogramming. With over 10000 tumors comprising 33 tumor types, Thorsson et al identified 6 immune subtypes based on immunogenomic analysis (**Table 2**). Additionally, these 6 immune subtypes also presented different somatic aberrations 33. Another study leveraged The Cancer Genome Atlas (TCGA) dataset's matched genomic and transcriptomic information to perform extensive analysis. The authors not only recapitulated the above classification but also added a complementary of those 6 subsets, which mostly includes leukocytes. Further variation analysis revealed that either common or rare germline variants are associated with differential immune cell infiltrations and immunomodulatory pathways 34. For example, common single-nucleotide polymorphisms (SNPs) in both IFIH1 and TMEM173 (STING) are strongly associated with IFN signaling. A rare germline mutation in COL7A1 corresponds to an increase in macrophage infiltration and a decrease in lymphocyte infiltration 34.

At a deeper level, tumor heterogeneity is one of the major causes of antigenic response, whereas protein coding gene mutations and indels are the main sources of tumor neoantigens 35. Thus, as genomic tumor heterogeneity increases, so does the heterogeneity of neoantigens 36. Tumor heterogeneity influences TME immunity through diverse neoantigens. Germline mutations in mismatch repair (MMR) genes affect the somatic mutation rate and neoantigen load 34. Dying tumor cells release neoantigens, which recruit different kinds of immune cells. Subsequently, the patterns of immune cell infiltration are diverse, including immune cell composition, immune cell distribution (infiltrating in tumors or scattering surrounding the tumor), and immune cell function (effector T cells or dysfunctional T cells) 37. In summary, tumor heterogeneity acts as the source of dynamic TME reprogramming and the cause of TME complexity during tumor progression. As a crucial mediator of tumor development and treatment, the TME deserves more attention and research.

## The role of tumor heterogeneity in drug resistance

During tumor progression and development, clonal evolution and tumor heterogeneity lead to a series of biological and host environment changes, mainly through the alteration of transcriptome expression and crosstalk among cells. Tumor heterogeneity itself and these changes lead to drug resistance, which is evident in all cancer types and treatment modes. Here, we describe key mechanisms through which tumor heterogeneity causes drug resistance upon molecularly targeted therapy and immunotherapy in the TME reprogramming contexture.

### Tumor heterogeneity impacts on therapeutic targets

The expression of therapeutic targets is variable and subjected to continuous change during tumor development. Stewart et al. conducted a study that focused on determining the underlying mechanisms by which small-cell lung cancer (SCLC) evolves rapidly from chemosensitivity to chemoresistance 38. An SCLC circulating tumor cell (CTC)-derived xenograft (CDX) model was generated to mimic patient tumor platinum-sensitive and platinum-resistant responses. scRNA-seq was applied to generate transcriptome data for chemosensitive and chemoresistant CDXs and CTCs, and a great increase in intratumor heterogeneity was identified in the resistance model. To determine the association between intratumor heterogeneity and drug resistance, the authors performed extended treatment for platinum-sensitive CDX models with cisplatin chemotherapy until relapse. Herein, they confirmed that relapse was related not only to increased intratumor heterogeneity but also to variations in the expression of therapeutic targets (DLL3, AURKA/AURKB, PARP1, MYC, BCL2, KDM1A, TOP1, TOP2A, VEGFA) and EMT genes within the same patients 38. The complexity of the transcriptome exerts a profound effect on drug responses. Mechanically, for example, DLL3 expression is variable and dynamic in SCLC, which means that DLL3-positive and DLL3-negative cells coexist in SCLC. The DLL3 expression heterogeneity contributes to resistance or low response rates even selecting for the high expression level of SCLC patients. Additionally, with treatment, the authors found that DLL3 expression may dynamically decrease and disappear. For other therapeutic targets, such as AURKA/AURKB and PARP1, this phenomenon also exists. In addition, another study performed whole genome sequencing (WGS) of non-small-cell lung cancer CTCs resistant to anaplastic lymphoma kinase (ALK)-tyrosine kinase inhibitors (TKIs) and found that the CTCs showed wide copy number alteration (CNA) heterogeneity and elevated chromosomal instability (CIN). A total of 121 CNA oncogenic drivers were classified across patients in different signaling pathways. Among them, the cell cycle and DNA repair pathways were dominantly activated, as were the RTK/RAS, PI3K, and MYC pathways. Regarding the mechanisms of ALK-TKI resistance, ALK-negative CTCs activate bypass signaling pathways to drive resistance, while ALK-rearranged CTCs might drive resistance through epithelial-to-mesenchymal transition 39. In addition, Roper et al. described the resistance mechanisms of osimertinib-treated EGFR mutants in lung adenocarcinoma patients. They conducted multiregion/temporal exome and transcriptome data analysis, and their results indicated that the majority of patients had two or more osimertinib-resistance mechanisms, and amplification of mutant EGFR was found in 67% (n=8/12) of patients 40. Thus, clonal heterogeneity and tumor evolution contribute to acquire target drug resistance by variable and changed therapeutic targets.

## Tumor heterogeneity impacts on immunotherapy

### Cytotoxicity and frequency of tumor infiltrating leukocytes (TILs) are shaped by tumor heterogeneity

The cytotoxicity and frequency of TILs are shaped by tumor heterogeneity, which, in turn, affects immunotherapy. Ma and colleagues obtained the single-cell transcriptomic landscape of liver cancer biospecimens from 19 patients 41. They found that hepatocellular carcinoma (HCC) and intrahepatic cholangiocarcinoma (iCCA) present different levels of transcriptomic heterogeneity. To better define the level of heterogeneity, the authors developed a method to compute a diversity score for each tumor and divided samples into diversity-high (Div-High) and diversity low (Div-low) groups. They also found that Div-High group patients tend to obtain aggressive tumor features and present a poor prognosis. Hereafter, the authors investigated whether Dive-High tumor cells produce cellular factors to induce TME reprogramming. They first performed Ingenuity Pathway Analysis (IPA) 42 to search for upstream regulators of each nonmalignant cell type (CAFs, TAMs, TECs, T cells). Then, they selected the upstream cytokine/growth factor that was found in at least 3 of 4 cell types. Finally, they overlapped these selected genes with the differentially expressed genes between Div-High and Div-Low malignant cells as candidate genes. Here, vascular endothelial growth factor A (VEGFA) is one of the top candidate genes. In the literature, VEGFA is a direct target of hypoxia-inducible factor 1-alpha (HIF1A), which is the key factor in sensing hypoxia 43. Furthermore, they found that hypoxia-related genes, as well as HIF1A, were expressed at higher levels in the Div-High group than in the Div-Low group. Thus, the results demonstrated that hypoxia-induced VEGF in the Div-High group triggers the polarization of TME stromal cells (CAFs, TAMs, TECs). On the other hand, the transcriptomic profiles of T cells from Div-Low and Div-High were completely different. Further analysis of gene set enrichment revealed that EMT and myogenesis pathways were upregulated in Div-High T cells. Additionally, in Div-Low tumors, T-cell cytotoxicity-related genes were upregulated, which means that in tumors with higher heterogeneity, T cells showed lower cytolytic activities. Conversely, the frequency of Treg cells is much higher in Div-High cells. Taken together, tumor heterogeneity drives TME reprogramming both through stromal cell polarization and changing T-cell cytolytic activities and ultimately influences the therapeutic outcome. In another study, Samstein et al. identified that mutations in two homologous recombination genes, BRCA1 and BRCA2, have a profound impact on the TME and then divergently affect immunotherapy, where BRCA1 deficiency results in ICB resistance, but BRCA2 deficiency is associated with an improved response 44. To further explore the mechanism underlying this phenomenon, the authors found that mutations in these two genes modulate distinct genomic alteration patterns and gene expression, which lead to divergent immune microenvironments. More detailed analysis revealed that the infiltration proportion of Cd4^+^, Cd8a^+^ T cells, NK cells, myeloid cells, and dendritic cells and the expression of some cytotoxicity genes were different in Brca1^null^ and Brca2^null^ tumors. Overall, Brca2^null^ tumors are more likely to present T-cell phenotypes, while Brca1^null^ tumors tend to be associated with an immunosuppressive TME, which suppresses the cytotoxic T-cell effect. Tumor-infiltrating myeloid cells (TIMs) play important roles in regulating tumor progression and therapy responses and consist of several distinct lineages, including dendritic cells, monocytes, macrophages, neutrophils, mast cells and myeloid-derived suppressor cells (MDSCs) 45. To some extent, the TIM compositions are shaped by distinct somatic mutations and transcriptomic patterns 46. Some of these TIMs can lead to drug resistance and poor survival rates. For example, the elevated MDSCs were related to reduced TILs and cytolytic function, which results in ICB resistance 47, 48. To uncover the mechanism, de Henau et al. compared multiple mouse models that were treated with ICB to determine the associations between ICB resistance and myeloid cell infiltration. Resistance to anti-PD-1 or anti-CTLA-4 therapy was found in the 4T1 breast cancer mouse model accompanied by enriched myeloid cells (CD11b+) also known as MDSCs. However, in the B16-F10 melanoma model, the ICB response presented well and enriched more activated CD8^+^ T cells and fewer myeloid cells. When transduced with the MDSC recruitment factor granulocyte-macrophage colony-stimulating factor (GM-CSF) into a B16-F10 melanoma model, ICB sensitivity was lost, which indicates the pivotal role of MDSCs in ICB therapy resistance 48.

### The heterogenous expression of cancer immunity genes impact immunotherapy

Drug resistance in tumor immunotherapy could be induced by changes in the expression of cancer immunity genes triggered by intratumor heterogeneity. The genes involved in immunotherapy could participate directly or indirectly. For example, immune checkpoint inhibitor (ICI) therapy is the most representative form of immunotherapy. Currently, there are three groups of American Food and Drug Administration (FDA)-approved ICIs, including cytotoxic T lymphocyte-associated protein 4 (CTLA-4), programmed cell death 1 (PD-1) on T cells, and PD-L1 on tumor cells 49. However, only a small percentage (20-30%) of patients has positive responses to PD1 and/or PD-L1 therapy 50. The intensity of immunity gene expression could directly impact resistance to immunotherapy 51. PD-L1 expression presents a heterogeneous paradigm in tumor cells 52. Thus, tumors without or with low expression of PD-L1 tend to generate anti-PD1 and anti-PD-L1 resistance. This assertion is supported by the overall response rates (ORRs) of patients with high and low PD-L1 expression. The response rates were only approximately 10% in PD-L1 low expression melanoma patients, while the percentage increased to approximately 40-50% in their PD-L1 high expression counterparts. Similar responses also occurred in other tumor types, such as non-small-cell lung cancer 53 and SCLC 54. In addition, the expression of PD-L1 in immune cells is associated with IFNγ-induced adaptive regulation and lymphocyte and effector T-cell infiltration 55. However, as JAK/STAT signal induction involves PD-L1 expression 56, 57, genetic alteration of genes related to this signaling pathway may impact PD-L1 expression. The loss of PTEN, the mutation of PI3K, AKT, EGFR, and the overexpression of MYC could also upregulate the PD-L1 expression level 58. Other resistance mechanisms also emerge. For example, the loss of PTEN could promote the expression of immunosuppressive cytokines such as VEGF to lead to reduced T-cell infiltration and autophagy inhibition, which decreased T-cell-mediated tumor killing 59.

### Tumor heterogeneity leads to activation or loss of key signaling pathways in tumor cells

Interferon-γ (IFN-γ) is secreted mainly by CD8^+^ T cells, CD4^+^ T cells, and NK cells, which have been reported to mediate tumor resistance by regulating core immune checkpoint proteins and chemokines to exert anti-proliferative and pro-apoptotic effects on cancer cells 60, 61. JAK1 and JAK2 signaling are essential factors that contribute to the success of immunotherapy. Loss-of-function mutations in these two genes result in immune checkpoint blockade resistance by downregulating PD-L1 expression and then responding to IFN-γ signaling 62. Another study also reported that truncated mutation in these two genes led to a lack of response of IFN-γ, which results in insensitivity to its anti-proliferation of cancer cells 63.

Jason et al. used a CRISPR/Cas9 genome editing screen to identify PD-1-resistant mutants (Ifngr2 and Jak1) in B16.SIY melanoma cells, and they confirmed the importance of IFN-γR signaling in the resistance process *in vitro*, which plays a great role in the T-cell-mediated tumor cell killing process 64. However, when IFN-γ signaling mutant tumor cells were implanted into mice, the antitumor response was improved, and CD8^+^ T cells were validated to be responsible for this improvement. More detailed analysis revealed that defective IFN-γ signaling in tumor cells leads to an increase in tumor antigen-specific CD8^+^ T cells in the TME. To investigate the discrepant phenomena that exist *in vivo* and vitro, the authors suggested the following two model systems. First, due to tumor heterogeneity, a minor subset of IFN-γ signaling-mutant tumor cell clones was selected. Second, the PD1 antibody might neutralize the negative effect of PD-L1 on WT tumor cell clones. To test this hypothesis, the authors inoculated the IFNγR2 mutant together with WT tumor cells into mice, which was used to mimic human patient heterogeneity. The results showed that tumors grow slowly but progressively, which suggests that the WT tumor cells provide missing negative regulatory signals. After being treated with PD-L1 antibody, the tumor escaped, and the IFNγR2-mutant tumor cells indeed proliferated. As in the context of PD-L1 therapy, the antiproliferative effects might not occur, and the IFN-γ dominated antitumor effects may emerge. Therefore, the growth of IFN-γ-insensitive tumor cells depends on PD-L1 expression by WT tumor cells. For these phenomena, the authors suggested that heterogeneity in the expression of mutant antigens could drive dominant T cells to kill cancer cells under immunotherapy. Altogether, the results demonstrate that tumor heterogeneity and clonal cooperation can partially produce immunotherapy resistance 64.

This IFN-γ signaling cascade-related genetic evolution resistance can also evolve into T-cell-resistant HLA class I-negative lesions with antigen presentation genes silenced and insensitive to IFN-γ signaling 65. In addition, Yu et al identified another mechanism by which IFN-γ signaling induces tumor resistance. IFN-γ facilitates nuclear translocation and YAP phase separation, and tumor cell YAP condensation is promoted, which forms a transcription hub to maximize target gene transcription to modulate anti-PD-1 immunotherapy resistance 66.

### Tumor heterogeneity determines the abundance of neoantigens to affect immunotherapy

Neoantigens are those antigens produced by tumor cells that are induced by genetic mutations, aberrant alternative splicing, and other reasons. Genetic mutation is the main source of neoantigen. Mutations that occur in coding and noncoding regions lead to aberrant amino acid sequences to synthesize abnormal peptides or proteins in tumor cells. These aberrant peptides or proteins can be recognized by the immune system and trigger immune responses, which is another target of immunotherapy 67, 68. Tumor heterogeneity leads to variable expression of neoantigens 69, which directly affect the abundance and types. Wolf et al. focused on the effect of tumor mutation burden (TMB) and intratumoral heterogeneity on tumor aggressiveness and their impact on antitumor immune responses in melanoma 70. To uncouple TMB and intratumor heterogeneity, the authors established an *in vivo* mouse system with ultraviolet radiation b(UVB) irradiation exposed to the B2905 melanoma cell line. First, they irradiated the cells with UVB (generated increased heterogeneity and mutation load tumor cells), and then single-cell-derived clones (generated decreased heterogeneity and random mutation load tumor cells) were extracted from UVB-treated cells. When wild-type (WT) C57B/6 mice were inoculated with parental B2950 cells, UVB-irradiated cells, and single-cell-derived cells, the tumor growth rate and aggressiveness displayed significant differences. While inoculated in Cg-Prkdc^scid^Il2rgt^m1Wjl/SzJ^ (NSG) immunocompromised mice (T, B, and NK cells defective), there was no difference in tumor growth, which indicated that the immune system should be responsible for this phenomenon. Further experiments and analysis showed that the decreased growth rate of single-cell-derived cells (low intratumor heterogeneity) is due to elevated T-cell reactivity and tumor infiltration. Two tumor heterogeneity fundamental components, the number of clones and the diversity of mutations, have been validated and confirmed to be determinants of tumor growth. Moreover, they found that tumors with high levels of these two components showed poorer survival in cohorts who had undergone checkpoint inhibitor therapy. Based on the above results, the authors suggested that mechanically, low heterogeneity tends to reduce the landscape of neoantigens and expose reactive neoantigens to immune detection. Then, the increased immune cells infiltrate to tumor core, and more antigens are presented in the TME, which enhances the immune response to reject tumor growth. In contrast, a high level of heterogeneity would “dilute” the reactive neoantigens, which leads to a strong immunosuppressive tumor microenvironment, manifested by reduced tumor immune cell infiltration, cytotoxicity, and effector cytokine secretion 70. Furthermore, Lorenzo et al. 71 illustrated that immune infiltrated cell type, density, location, and functional orientation in the tumor microenvironment elicit prominent impacts on anticancer efficacy. Hence, according to research and theory, tumor heterogeneity greatly affects the therapy response and outcome. For precision medicine in this area, assessing tumor heterogeneity and its microenvironment condition might be necessary.

## Technologies to explore tumor heterogeneity

To date, several technologies, such as liquid biopsy 72-74, single-cell sequencing, spatial transcriptomics sequencing, and spatial genomics sequencing, have been successfully applied to evaluate and explore tumor heterogeneity.

### Liquid biopsy

Liquid biopsy is a method to noninvasively collect cancer samples in various body fluids instead of a fragment of cancer tissue. It allows people sampling at different time points to monitor and analyzing tumor heterogeneity, and track tumor evolution dynamics with various analysts, such as circulating tumor cells, cell-free DNA, cell-free RNA, exosomes, and tumor-educated platelets 75.

### Single-cell sequencing

Single-cell sequencing can be used for transcriptomic characterization 76, cell type identification 76, clonal decomposition and DNA replication state definition 77, characterization of cancer ecosystem features and their associations with clinical data 78 and the identification of novel or potential cancer therapeutic targets and strategies (**Table 3**).

### Spatial transcriptomics sequencing

**Figure 3:**
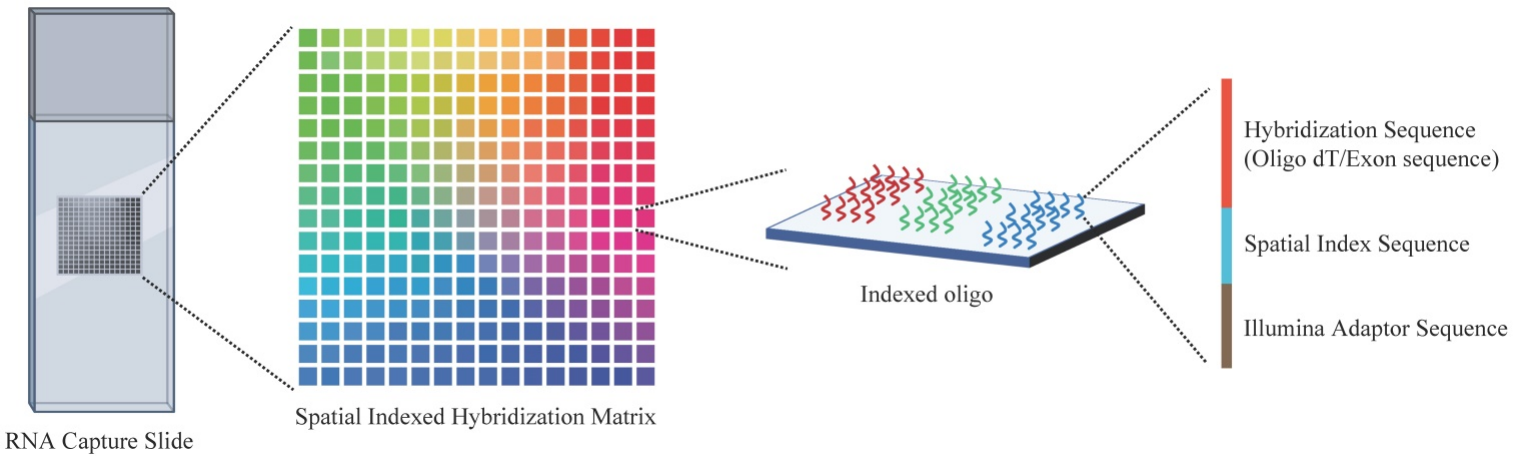
Overview of the spatial transcriptome sequencing capture slides. Each slide contains millions of spatially indexed hybridization-oligo clusters, which allow the capture of released tissue mRNA *in situ*, enabling the dissection of gene expression information. Specifically, the tissue section was fixed and permeabilized to release mRNA. Then, mRNA binds to indexed hybridization oligo clusters. After that, the captured mRNA is synthesized into cDNA and liberated from the slide for further sequence library preparation.

Spatial transcriptomics is an overarching term for a range of methods that could anchor the gene expression to their locations, with a single cell level or multicellular area, in the histological sections (**Figure 3**). It is essential to decipher the intratumor spatial heterogeneity.

Although the development of scRNA-seq technology has led to new insights into tumor heterogeneity, how tumor tissues functional organization and cell-cell crosstalk *in situ* still untouched. Therefore, there is a desperate need to understand the spatial distribution and the intrinsic biological meaning. To this end, researchers have developed microdissection-based small bulk samples or single-cell capture at a particular location to check their gene expression or fluorescent *in situ* hybridization (FISH)-based technologies to illustrate diverse gene features *in situ*. However, both approaches are restricted by limited throughput, which only involves a small number of cells or a few expressed genes at a time. In 2016, with the help of Stahl et al., spatial transcriptomics technology was established. This method used individual cells on the array of spatially barcoded reverse transcription primers that are able to capture mRNA with oligo (dT) tails 79. In silico reconstruction of spatial gene expression patterns allows visualization and quantitative analysis of the transcriptome with spatial resolution in individual tissue sections.

With the help of spatial transcriptomics sequencing, Emelie et al. plotted the spatial maps of prostate cancer and identified gene expression gradients in stroma adjacent to tumor regions that allow for re-stratification of the tumor microenvironment 80. This indicates that this newly developed technology could help people successfully reveal the unexplored landscape of heterogeneity. Then, it was widely used in other types of tumors, including melanoma 81, neuroblastoma 82, gastric cancer 83, and breast cancer 83. In the effort of invasive micropapillary carcinoma (IMPC) studies, spatial transcriptomics analysis provides a valuable resource for exploring the inter- and intratumoral heterogeneity of IMPC and identifies a new marker, SREBF1, which facilitates accurate diagnosis and treatment of the disease 83. The spatial heterogeneity analysis-guided treatment decision approach has also been further validated in other types of tumors 84, 85. Recently, a group from the Kinghorn cancer center established a single-cell and spatially resolved atlas for human breast cancers, which includes both scRNA-seq and spatial transcriptomics of more than 20 patients. This work demonstrated high-resolution neoplastic cell heterogeneity and immune profiles and further deconvoluted large breast cancer cohorts to stratify them into nine clusters, termed ecotypes. This study provides a comprehensive transcriptional atlas of the cellular architecture of breast cancer.

### Spatial genomics sequencing

Compared to spatial transcriptomics sequencing, spatial genomics sequencing supplies more direct information to delineate intratumor genetic heterogeneity, which could not only reveal how tumor clones are organized but also uncover to what extent that tumor progression is driven by genetic alterations and the TME. Recently, Zhao and colleagues developed a method called slide-DNA-seq, which could capture spatially resolved DNA sequences from intact tissue 86. The core of this method is that they can fragment and barcode DNA *in situ* to preserve accurate local tumor architecture information, which enables the discovery of clonal populations and their spatial regions. In their study, the spatial distribution of copy number alterations and distinct tumor clones were characterized. In addition, when integrated with transcriptomics sequencing, the authors dissected how genetics and TME control the transcriptional program. Thus, by adding spatial information, spatial genomics sequencing is a promising method in cancer clonal heterogeneity research.

## Strategies to overcome tumor heterogeneity-induced drug resistance

### Combination therapy

Targeting tumor heterogeneity is one of the potential approaches to combat tumors. Combination therapies with more than one therapeutic mechanism can directly target the pre-existing and post-emerging subpopulations or prevent resistant subclone selection. For example, in HER2^+^ breast cancer patients, both the expression and copy number alteration of HER2 vary, and the high level of tumor heterogeneity for HER2 responds poorly to monotherapy 87, 88. However, by using combined chemotherapy and phosphatidylinositol 3-kinase/AKT inhibitor treatment in the high level of HER2 heterogeneity, the efficacy was markedly improved. Additionally, in a meta-analysis study, combined chemotherapy and immunotherapy samples presented improved clinical results in NSCLC 89. For some acquired drug resistance, such combination therapy was also researched and applied. In EGFR-mutant lung adenocarcinoma, after treatment with tyrosine kinase inhibitors (TKIs), the majority of patients acquired subclonal drug-resistant mutations, such as MET, PD-L1, KRAS amplification, ESR1-AKAP12, and MKRN1-BRAF fusions. Thus, based on the results, combination therapies were suggested to overcome this acquired resistance to block clonal outgrowth 40.

The application of combination therapy in the immunotherapy area has also been effective. For example, MDSCs have been found to correlate with decreased CD8^+^ T-cell infiltration and cytolytic function 48. To avoid and overcome the effect of MDSCs on ICB treatment, people have tried to combine ICB with targeting MDSCs to suppress or eliminate the activity of MDSCs and enhance ICB therapy efficacy. Because phosphoinositide 3-kinase (PI3K-γ) is upregulated in myeloid cells, PI3K-γ was selected and inhibited by a PI3K-γ inhibitor to neutralize MDSC-associated ICB resistance 48. Similar therapy was performed on metastatic castration-resistant prostate cancer patients, and the efficacy was significantly improved 47. In this design, the authors used the multikinase inhibitors cabozantinib (tyrosine kinase inhibitors) and BEZ235 (PI3K/mTOR dual inhibitor) to selectively deplete MDSCs. Mechanically, the combination of ICB and MDSC-targeted therapy can facilitate prostate cancer cells to upregulate IL-1ra to inhibit IL-1-induced MDSC chemoattraction and suppress MDSC-promoting cytokines.

Another specific combination therapy is called antibody-drug conjugates (ADCs), which have been proven to be an effective strategy for combating cancer cells across several tumor types 90-93. By September 2021, 11 ADCs acquired approval from the FDA 94. Most ADCs contain a cytotoxic agent and a monoclonal antibody that recognizes the tumor-associated antigen 95. Thus, the antibody, the cytotoxic drug (called payloads), and the linker connecting the payloads to the antibody are the three key elements of an ADC 96. Although people have achieved striking clinical success in ADC treatment, tumor heterogeneity frequently contributes to drug resistance through alterations in the targets and heterogeneous expression of targets 97, 98. Recently, Yamazaki et al. used a novel ADC, which contains two distinct payloads for combating HER2 heterogeneity and drug resistance 98. In the HER2+ heterogeneous breast xenograft model, this ADC presented great therapeutic potential, with enhanced efficacy and minimal inflammation.

### Three-dimensional tumor slice culture (3D-TSC) platform

**Figure 4:**
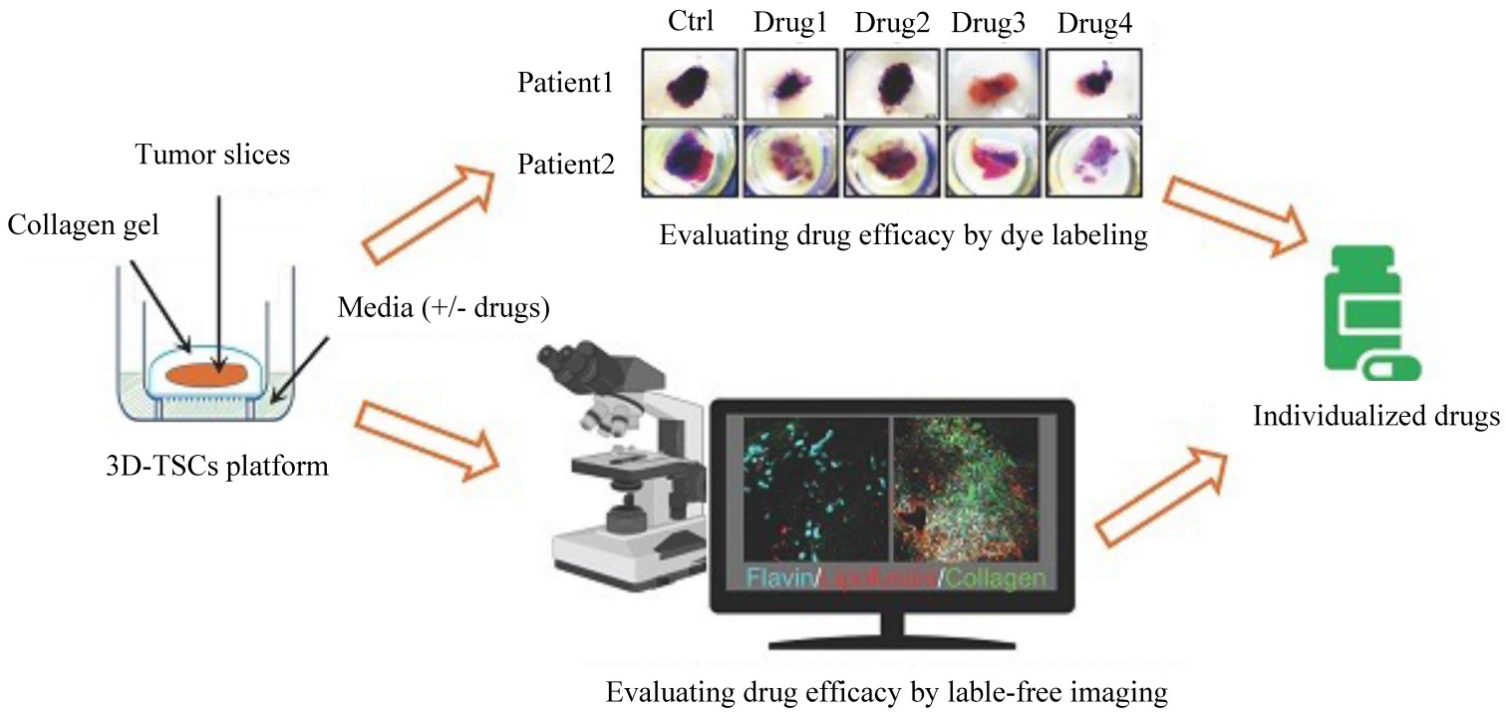
Schematic diagram of the three-dimensional tumor slice culture (3D-TSC) platform and workflow for precise anticancer drug discovery. Tumors were biopsied, and thick tissue slices (300 µm) were prepared by using a Leica VT1200 S vibratome (Leica Biosystems Nussloch GmbH, Germany) within 2-6 h after surgery. The thick slices were then cultured on a 3D-TSC platform. To screen sensitive and efficient anticancer drugs, the cultured tissue slices were treated with different drugs. Then, two evaluation systems, a dye labeling assay and label-free fluorescence imaging, were performed to monitor and evaluate tumor cell apoptosis and viability. In the dye labeling assay, 3-(4,5-dimethylthiazol-2-yl)-2,5-diphenyltetrazolium bromide (MTT) or propidium iodide (PI) staining was used. After drug treatment, we evaluated tumor cell apoptosis based on the staining and fluorescence intensity. Compared to the dye labeling assay, label-free imaging could be performed at different time points by a Leica M165FC fluorescent stereomicroscope. The tumor cell apoptosis and viability results could be used to predict the drug efficacy and provide guidelines for the treatment of the corresponding patients.

Some novel and effective preclinical models could be implemented to assess drug responses and efficacy. For example, a three-dimensional tumor slice cultures (3D-TSC) platform (**Figure 4**) was reported recently, which combines histochemical staining, transgenic fluorescent reporter, and label-free metabolic imaging approaches in a time course to evaluate drug efficacy to accelerate precision drug screening for cancer therapy 99. As 3D-TSC not only maintains important original tumor features, such as tumor heterogeneity, gene expression, and cell architecture but also maintain all immune components, it should be a powerful model for testing the drug sensitivity.

## Conclusion and future perspective

In summary, tumor heterogeneity and tumor clonal evolution undergo dynamic changes and accompany the whole tumor development process. Tumor heterogeneity impacts the tumor ecosystem by modulating the gene expression of all components and interactions with components that exist in the TME to constantly reshape it, which plays significant roles in drug responses. As tumor heterogeneity influences drug responses in nearly all therapeutic modes and all types of cancer patients through different mechanisms, more efforts should be made to rethink therapeutic strategies. On the one hand, personalized medicine should be applied here to continually evaluate the dynamic changes in tumor heterogeneity levels and TME complexity levels to better choose more appropriate drugs for patients. On the other hand, some novel and effective preclinical models, such as the 3D-TSC platform, could be implemented to assess drug responses and efficacy 99. 3D-TSC is a good model for observing dynamic changes in the cell population during drug treatment. If gene expression profiling by scRNA-seq and spatial transcriptomics sequencing can be conducted, the model should facilitate the understanding of the spatial and temporal evolution of the TME during treatment and provide useful information for enhancing the efficacy of anticancer drugs. Since tumor heterogeneity has been a major trigger of drug resistance, future work should also aim to uncover the influence of genetic evolutionary processes on tumor heterogeneity, how tumor heterogeneity affects TME remodeling, and how it affects drug responses under the contexture of TME.

## Figures and Tables

**Table 1:** TNBC subtypes were classified by different molecular levels

Classification based	Subtype name	Signatures	References
Cell populations	Neutrophil-enriched (NES)	Chemo-attractants: TNFAIP6, CXCL1/2, CCR2	100
	Macrophage-enriched subtypes (MES)	Epithelial-mesenchymal related genes: Zeb1, Cdh1	
Immunogenomic	Immunity High	CORO1A, STAT4, BCL11B, ZNF831, EOMES, CD247, CD8A, MAP4K1	101
	Immunity Medium	IRF8 and SPI1	
	Immunity Low		
Gene expression	Basal-like1 (BL1)	Heavily enriched in cell cycle and cell division components and pathways	102
	Basal-like2 (BL2)	Growth factor signaling, glycolysis and gluconeogenesis	
	Immunomodulatory (IM)	Immune cell processes	
	Mesenchymal (M)	Cell motility, ECM receptor interaction, and cell differentiation pathways	
	Mesenchymal stem-like (MSL)	MSL subtype expresses low levels of proliferation genes	
	Luminal androgen receptor (LAR)	Hormonally regulated pathways including steroid synthesis, porphyrin metabolism, and androgen/estrogen metabolism; highest mutation burden in PIK3CA, KMT2C, CDH1 et al.	
Spatial patterns of CD8^+^ T cell localization and gene expression signatures	Margin-restricted (MR)	An accumulation of CD8+ T cells at the tumor margins	103
	Immune desert (ID)	A low abundance of CD8+ T cells at the margins	
	Fully inflamed (FI)	Significant CD8+ T cell infiltration into the tumor epithelial compartment	
	Stroma-restricted (SR)	CD8+ T cell accumulation in the stroma and exclusion from the tumor epithelial compartment	
Gene expression	Luminal androgen receptor (LAR)	Androgen receptor signaling; low chromosomal instability; CDKN2A/B loss (RB1 neutral)	104
	Immunomodulatory (IM)	High immune cell signaling and cytokine signaling gene expression; relatively high chromosomal instability	
	Basal-like immune-suppressed (BLIS)	Upregulation of cell cycle, activation of DNA repair, and downregulation of immune response genes; high chromosomal instability; frequent 9p23 and 12p13 amplification	
	Mesenchymal-like (MES)	Enriched in mammary stem cell pathways; copy-number profile between LAR and the other two groups	
Copy number alteration	Chr9p23 amp	Frequent 9p23 amplification	104
	Chr12p13 amp	Frequent 12p13 amplification	
	Chr13q34 amp	Frequent Chr13q34 amplifications	
	Chr20q13 amp	Frequent Chr20q13 amplification	
	Chr8p21 del	Frequent Chr8p21 loss	
	Low CIN	Low chromosomal instability	

**Table 2:** The 6 immune subtypes and the correspond features

Immune subtypes	Immune expression signatures
C1: wound healing	Angiogenic genes upregulated
	Tumor cell with high proliferation rate
	A Th2 cell bias to the adaptive immune infiltration
C2: IFN-γ dominant	Highest M1/M2 macrophage polarization
	Strong CD8 signal
	High level of TCR diversity
	Tumor cell with high proliferation rate
C3: inflammatory	Elevated Th17 and Th1 genes
	Tumor cell proliferation rate at low to moderate levels
	Lower level of aneuploidy and CNAs
C4: lymphocyte depleted	Significant macrophage signature
	Th1 suppressed
	A high M2 response
C5: immunologically quiet	Lowest lymphocyte
	Dominated by M2 macrophages
	Enriched IDH mutations (IDH mutations associate with TME composition 105)
C6: TGF-β dominant	High level of TCR diversity
	Highest TGF-β signature
	A high lymphocytic infiltrate with an even distribution of type I and type II T cells

**Table 3:** Summary of studies investigating novel or potential cancer therapy by single- cell RNA sequencing

Cancer type/model	Identified cell subsets	Potential target for therapy	Findings/Mechanisms	References
Colon cancer	C1QC^+^ TAM subset SPP1^+^ TAM subset	Anti-CSF1R blockade (tumor associated macrophages (TAMs))	Distinct sensitivity of two macrophage populations to anti-CSF1R (sensitive to C1QC^+^ TAM subset, sparing SPP1^+^ TAMs)	106
	Conventional DC1 (XCR1/BATF3 positive DCs)	Anti-CD40 agonist (Dendritic cells)	Increases Effector Memory CD8^+^ T Cells & induces Bhlhe40+ Th1-like Cells activation and proliferation	
Breast cancer	FAP^+^/CAF-S1's ecm-myCAF subset (one of the eight FAP^+^/CAF-S1 cluster)	Combining PD-1 and or/CTLA4 blockade with targeting ecm-myCAF	Increase the PD-1 and CTLA4 protein levels of FOXP3^+^ T cells (Treg)	107
Cholangiocarcinoma	Granulocytic myeloid-derived suppressor cells (G-MDSCs)	Dual inhibition of TAMs and granulocytic myeloid-derived suppressor cells (G-MDSCs) potentiated anti-PD-1 therapy	TAM blockade failed to decrease tumor progression due to a subset G-MDSCs compensation. However, when inhibit this G-MDSCs, the ICB therapy efficacy improved	108
Intrahepatic cholangiocarcinoma (ICC)	Vascular cancer-associated fibroblasts (vCAFs)	Interleukin (IL)-6/IL-6 receptor (IL-6R)	IL-6 that secreted by vascular cancer-associated fibroblasts (vCAFs) upregulated enhancer of zeste homolog 2 (EZH2) to promote tumor progression	109
Osteosarcoma (OS)	CD8+ T, CD4+ T, and NKT cells	CD8+ T, CD4+ T, and NKT cells widely expressed TIGIT	Blocking TIGIT substantially enhanced the death of OS cells that triggered by CD3+ T cells derived from relatively high TIGIT^+^CD3^+^ T cells infiltration in OS tissues	110
Melanoma	cytotoxic CD8+ T cell subpopulation	PMEL, TYRP1, and EDNRB	These three genes were upregulated in exhausted cytotoxic CD8+ T cell subpopulation.	111
Ovarian cancer (organoid culture)	NK cells and a subset of CD8 T cells	bromodomain-containing protein BRD1	BRD1 inhibition could enhance PD1 and PD-L1 immune checkpoint blockade by decreasing NK cell and a subset of T cell exhaustion	112
Ovarian cancer	T cell and tumor cell	CXCL16-CXCR6	CXCL16 is responsible for T cell recruitment and was highly expressed by tumor cells. Its receptor CXCR6, was highly expressed by dysfunctional CD8+ GZMB T cells and CD4+ FOXP3 regulatory T cells (Tregs)	113
Bladder cancer	LAMP3^+^ DC subgroup	CCL17, CCL19, and CCL22 were expressed by LAMP3^+^ DCs	LAMP3^+^ DCs was responsible for Tregs and CCR4^+^ cells recruitment	114
	Inflammatory cancer-associated fibroblasts (iCAFs)	CXCL12, VEGFA, VEGFB, FGFR1 were expressed by iCAFs	iCAFs expressed CXCL12, VEGFA, VEGFB, FGFR1 and interacted with endothelial cells and immune cells to facilitate angiogenesis and immune suppressiveness to promote tumor proliferation	
Clear-cell renal cell carcinoma (ccRCC)	exhausted CD8^+^ T cells	LAG3 and HAVCR2	LAG3 and HAVCR2 (TIM3) were higher expressed in exhausted CD8^+^ cells than that PD-1 expressed	115
Small cell lung cancer (SCLC)	PLCG2-high tumor cell	PLCG2	PLCG2-high tumor cells are correlated with tumor metastasis, CD8^+^ T cell exhaustion, pro-fibrotic and immunosuppressive TME	116
Gallbladder cancer (GBC)	Epithelial cell subtype 1 and 2	ErbB pathway mutations	Epithelial cell subtype 1 and 2 with high level of ErbB pathway mutation, which secreted high level of midkine (MDK). Then MDK interacted with tumor-infiltrating macrophages's MDK receptor LPP1 to form immunosuppressive TME	117
